# Supplementary material for: Serum and Soleus Metabolomics Signature of Klf10 Knockout Mice to Identify Potential Biomarkers
Source: Metabolites. 2022 Jun 17;12(6):556. doi: 10.3390/metabo12060556 (PMC9231117; doi:10.3390/metabo12060556)
Supplement: Supplementary file 1 [file metabolites-12-00556-s001.zip › metabolites-1764790-supplementary.pdf]

Supplementary data

Table S1

Metabolite VIP	VIP value	Contribution score	p-value	Fold Change	Chemical classes
**PE(20:4(5Z,8Z,11Z,14Z)/18:1(11Z))**	1.524	2.07	$1.42\ 10^{-3}$	3.428	Phospholipids
**PE(22:6(4Z,7Z,10Z,13Z,16Z,19Z)/18:1(11Z))**	1.463	1.73	$3.24\ 10^{-3}$	3.738	Phospholipids
**Indoleacetic acid**	1.450	2.20	$1.77\ 10^{-4}$	2.569	Aromatic compounds-Amines
**Hippuric acid**	1.435	-2.44	$3.50\ 10^{-6}$	0.056	Amino acids-Derivatives
**PE(16:0/20:4(5Z,8Z,11Z,14Z))**	1.323	1.93	$3.91\ 10^{-4}$	2.151	Phospholipids
**Serotonin**	1.317	-2.15	$8.87\ 10^{-6}$	0.476	Amino acids-Derivatives
**Cholic acid**	1.301	2.01	$3.19\ 10^{-5}$	20.144	Lipids: Fatty acids-Steroids
**PC(14:0/16:0)**	1.270	1.98	$3.45\ 10^{-5}$	1.566	Phospholipids
**Guanidoacetic acid**	1.256	1.87	$9.68\ 10^{-5}$	2.290	Amino acids-Derivatives
**N-Acetylserotonin**	1.255	-1.94	$4.57\ 10^{-5}$	0.377	Amino acids-Derivatives
**L-Acetylcarnitine**	1.236	1.89	$6.81\ 10^{-5}$	1.531	Lipids: Fatty acids-Steroids
**O-Acetyl-L-carnitine**	1.233	1.88	$7.14\ 10^{-5}$		Lipids: Fatty acids-Steroids
**Glycerophosphocholine**	1.229	-1.75	$2.54\ 10^{-4}$	0.425	Lipids: Fatty acids-Steroids
**PC(15:0/20:2(11Z,14Z))**	1.223	1.82	$1.18\ 10^{-4}$	1.521	Phospholipids
**N-acetyltryptophan**	1.218	1.79	$1.52\ 10^{-4}$	2.990	Amino acids-Derivatives
**4-Trimethylammoniobutanoic acid**	1.213	1.82	$1.15\ 10^{-4}$	1.553	Lipids: Fatty acids-Steroids
**Trigonelline**	1.203	-1.79	$1.39\ 10^{-4}$	0.425	Aromatic compounds-Amines
**N-Methyltryptamine**	1.192	-1.61	$1.45\ 10^{-3}$	0.323	Aromatic compounds-Amines
**Tetradecanoylcarnitine**	1.176	1.68	$2.89\ 10^{-4}$	2.052	Lipids: Fatty acids-Steroids
**Cytosine**	1.164	-1.68	$3.10\ 10^{-4}$	0.601	Nucleotides-Nucleosides-Derivatives
**Nicotinic acid**	1.152	1.62	$6.80\ 10^{-4}$	1.659	Aromatic compounds-Amines
**L-Threonine**	1.149	-0.34	>0.05	0.964	Amino acids-Derivatives
**Ketoleucine**	1.148	-1.60	$7.24\ 10^{-4}$	0.474	Amino acids-Derivatives
**PC(15:0/16:0)**	1.138	1.57	$5.84\ 10^{-4}$	1.441	Phospholipids
**Cholesteryl acetate**	1.137	1.56	$1.10\ 10^{-3}$	1.929	Lipids: Fatty acids-Steroids
**L-Lysine**	1.136	-1.55	$6.95\ 10^{-4}$	0.780	Amino acids-Derivatives
**L-Valine**	1.128	1.24	$9.30\ 10^{-3}$	1.411	Amino acids-Derivatives
**Biotin**	1.099	1.49	$8.85\ 10^{-4}$	2.284	Vitamins-Cofactors-Hormones

**Uridine**	1.084	-1.39	1.82 10^{-3}	0.671	Nucleotides-Nucleosides-Derivatives
**Thiamine monophosphate**	1.084	1.42	2.17 10^{-3}	1.809	Vitamins-Cofactors-Hormones
**PC(O-18:2(9Z,12Z)/2:0)**	1.081	-1.45	1.16 10^{-3}	0.843	Phospholipids
**Oxoglutaric acid**	1.075	1.39	1.63 10^{-3}	1.702	Organic acids
**LysoPC(20:2(11Z,14Z))**	1.070	-1.42	1.37 10^{-3}	0.856	Phospholipids
**L-Serine**	1.069	0.02	>0.05	1.003	Amino acids-Derivatives
**D-Glyceraldehyde-3-phosphate**	1.066	-0.14	>0.05		Others
**3-(2-Hydroxyphenyl)propanoic acid**	1.059	1.39	1.84 10^{-3}	1.821	Aromatic compounds-Amines
**Indole-3-methyl acetate**	1.057	-1.39	1.72 10^{-3}	0.421	Aromatic compounds-Amines
**L-Pipecolic acid**	1.056	-1.38	1.86 10^{-3}	0.748	Amino acids-Derivatives
**Deoxycytidine**	1.052	-1.37	1.96 10^{-3}	0.671	Nucleotides-Nucleosides-Derivatives
**3-Methylhistidine**	1.045	-1.35	2.19 10^{-3}	0.768	Amino acids-Derivatives
**N-Alpha-acetyllysine**	1.038	-1.34	2.13 10^{-3}	0.794	Amino acids-Derivatives
**Glycocholic acid**	1.034	1.22	4.92 10^{-3}	21.090	Lipids: Fatty acids-Steroids
**LysoPC(18:0)**	1.016	-1.22	4.35 10^{-3}	0.857	Phospholipids
**Platelet activating factor**	1.016	-1.22	4.50 10^{-3}	0.857	Phospholipids
**L-Arginine**	1.015	-1.14	7.97 10^{-3}	0.729	Amino acids-Derivatives
**Indoxyl sulfate**	1.011	-1.24	3.61 10^{-3}	0.539	Aromatic compounds-Amines
**L-Malic acid**	1.008	1.17	5.88 10^{-3}	1.634	Organic acids
**PE(O-16:1(1Z)/22:6(4Z,7Z,10Z,13Z,16Z,19Z))**	1.006	1.10	1.57 10^{-2}	1.720	Phospholipids
**Citramalic acid**	0.997	1.23	3.74 10^{-3}	1.355	Organic acids
**Propionylcarnitine**	0.995	1.11	8.34 10^{-3}	1.520	Lipids: Fatty acids-Steroids
**PC(18:1(9Z)/18:1(9Z))**	0.995	1.22	3.75 10^{-3}	1.289	Phospholipids
**LysoPC(16:0)**	0.979	-1.17	5.30 10^{-3}	0.910	Phospholipids
**Stearic acid**	0.977	-1.08	9.25 10^{-3}	0.707	Lipids: Fatty acids-Steroids
**Ornithine**	0.964	1.03	1.25 10^{-2}	1.479	Amino acids-Derivatives
**Corticosterone**	0.960	0.72	>0.05	1.488	Lipids: Fatty acids-Steroids
**Uracil**	0.958	-1.09	7.39 10^{-3}	0.706	Nucleotides-Nucleosides-Derivatives
**LysoPE(0:0/18:1(11Z))**	0.929	0.86	3.41 10^{-2}	1.434	Phospholipids
**PC(16:0/16:0)**	0.905	1.00	1.05 10^{-2}	1.276	Phospholipids
**D-Glucose**	0.896	-0.77	>0.05	0.806	Sugar
**L-Asparagine**	0.888	0.96	1.26 10^{-2}	1.288	Amino acids-Derivatives
**PC(20:4(5Z,8Z,11Z,14Z)/22:6(4Z,7Z,10Z,13Z,16Z,19Z))**	0.874	-0.86	2.40 10^{-2}	0.648	Phospholipids
**Glucosamine 6-phosphate**	0.868	-0.82	3.09 10^{-2}	0.738	Sugar

**L-Glutamine**	0.856	0.89	$1.96\ 10^{-2}$	1.186	Amino acids-Derivatives
**L-Histidine**	0.854	0.88	$1.89\ 10^{-2}$	1.164	Amino acids-Derivatives
**Dodecanoylcarnitine**	0.848	0.89	$1.74\ 10^{-2}$	1.969	Lipids: Fatty acids-Steroids
**3-Methylhistamine**	0.842	-0.81	$2.77\ 10^{-2}$	0.731	Amino acids-Derivatives
**N-Acetyl-L-phenylalanine**	0.837	0.78	$3.28\ 10^{-2}$	1.412	Amino acids-Derivatives
**Gluconic acid**	0.827	-0.81	$3.22\ 10^{-2}$	0.575	Sugar
**Alpha-Tocopherol**	0.826	0.84	$2.49\ 10^{-2}$	1.203	Lipids: Fatty acids-Steroids
**LysoPC(16:1(9Z))**	0.826	-0.82	$2.75\ 10^{-2}$	0.839	Phospholipids
**Fumaric acid**	0.822	0.71	$4.96\ 10^{-2}$	1.424	Organic acids
**PC(O-14:1/2:0)**	0.813	-0.78	$3.77\ 10^{-2}$	0.828	Phospholipids
**Palmitic acid**	0.800	-0.74	$3.56\ 10^{-2}$	0.687	Lipids: Fatty acids-Steroids
**PC(O-14:0/2:0)**	0.791	-0.69	$4.68\ 10^{-2}$	0.908	Phospholipids
**3-Hydroxybutyric acid**	0.791	0.77	$2.84\ 10^{-2}$	1.379	Organic acids
**PC(O-16:1(11Z)/2:0)**	0.768	-0.70	$3.89\ 10^{-2}$	0.874	Phospholipids
**Azelaic acid**	0.764	-0.70	$4.63\ 10^{-2}$	0.756	Lipids: Fatty acids-Steroids
**Uric acid**	0.761	-0.72	$3.60\ 10^{-2}$	0.759	Nucleotides-Nucleosides-Derivatives
**Isocitric acid**	0.756	0.70	$4.12\ 10^{-2}$	1.196	Organic acids
**LysoPC(20:0)**	0.753	-0.52	$1.49\ 10^{-1}$	0.694	Phospholipids
**Citric acid**	0.748	0.69	$4.38\ 10^{-2}$	1.187	Organic acids
**Methylglutaric acid**	0.742	-0.66	$4.79\ 10^{-2}$	0.835	Lipids: Fatty acids-Steroids
**Phosphorylcholine**	0.738	-0.67	$4.35\ 10^{-2}$	0.679	Others
**PC(O-18:4/2:0)**	0.724	0.64	$4.87\ 10^{-2}$	1.140	Phospholipids
**LysoPC(20:3(5Z,8Z,11Z))**	0.706	-0.62	$5.48\ 10^{-2}$	0.837	Phospholipids
**Linoleic acid**	0.673	-0.43	>0.05	0.778	Lipids: Fatty acids-Steroids
**Orotic acid**	0.662	-0.52	>0.05	0.753	Nucleotides-Nucleosides-Derivatives
**PC(15:0/18:2(9Z,12Z))**	0.654	0.50	$9.01\ 10^{-2}$	1.031	Phospholipids
**trans-Ferulic acid**	0.644	-0.39	>0.05	0.504	Others
**L-Lactic acid**	0.607	-0.45	>0.05	0.928	Organic acids
**PC(15:0/20:1(11Z))**	0.551	0.38	$1.45\ 10^{-1}$	1.140	Phospholipids
**Allantoin**	0.532	-0.30	>0.05	0.885	Others
**PC(14:0/20:1(11Z))**	0.531	0.22	$3.86\ 10^{-1}$	1.105	Phospholipids
**Malonate**	0.495	0.30	>0.05	1.191	Organic acids
**Urocanic acid**	0.447	0.03	>0.05	1.037	Amino acids-Derivatives
**L-Methionine**	0.434	-0.22	>0.05	0.949	Amino acids-Derivatives

Table S2

Metabolites VIP	VIP value	Contribution Score	p-value	Fold Change	Chemical classes
**PC(O-14:0/2:0)**	1.798	-2.785	$3.99\ 10^{-4}$	0.852	Phospholipids
**Cytidine**	1.596	-2.154	$9.09\ 10^{-4}$	0.803	Nucleotides-Nucleosides-Derivatives
**Cytosine**	1.455	-1.975	$3.18\ 10^{-3}$	0.754	Nucleotides-Nucleosides-Derivatives
**Guanosine**	1.380	-1.546	$2.63\ 10^{-3}$	0.602	Nucleotides-Nucleosides-Derivatives
**Uric acid**	1.377	-1.509	$4.43\ 10^{-3}$	0.777	Nucleotides-Nucleosides-Derivatives
**D-Glucose**	1.353	1.687	$4.11\ 10^{-2}$	1.329	Sugar
**Platelet activating factor**	1.343	1.531	>0.1	1.303	Lipids: Fatty acids-Steroids
**Caprylic acid**	1.297	1.662	$6.04\ 10^{-3}$	2.065	Lipids: Fatty acids-Steroids
**L-Lactic acid**	1.289	1.459	$1.19\ 10^{-2}$	1.158	Organic acids
**Uracil**	1.285	-1.366	$6.32\ 10^{-3}$	0.659	Nucleotides-Nucleosides-Derivatives
**Propionylcarnitine**	1.273	1.373	$9.18\ 10^{-3}$	1.498	Lipids: Fatty acids-Steroids
**PC(16:1(9Z)/22:2(13Z,16Z))**	1.254	1.250	>0.1	1.095	Phospholipids
**Biotin**	1.243	-1.488	$6.45\ 10^{-3}$	0.576	Vitamins-Cofactors-Hormones
**Uridine**	1.234	-1.189	$1.10\ 10^{-2}$	0.662	Nucleotides-Nucleosides-Derivatives
**Methylguanidine**	1.221	1.193	$1.57\ 10^{-2}$	1.399	Nucleotides-Nucleosides-Derivatives
**3-Hydroxybenzoic acid**	1.220	-1.223	$7.60\ 10^{-3}$	0.682	Aromatic compounds-Amines
**Guanine**	1.199	-1.081	$3.33\ 10^{-2}$	0.778	Nucleotides-Nucleosides-Derivatives
**LysoPC(18:0)**	1.183	1.130	>0.1	1.259	Phospholipids
**Inosine**	1.181	-1.050	$1.81\ 10^{-2}$	0.768	Nucleotides-Nucleosides-Derivatives
**L-Homoserine**	1.164	1.352	>0.1	1.202	Amino acids-Derivatives
**L-Aspartic acid**	1.156	-0.991	$6.69\ 10^{-2}$	0.555	Amino acids-Derivatives
**PC(21:0)**	1.136	0.892	>0.1	0.984	Phospholipids
**Creatine**	1.133	1.035	$1.56\ 10^{-2}$	1.195	Amino acids-Derivatives
**PC(18:1(9Z)/18:1(9Z))**	1.123	1.024	>0.1	1.339	Phospholipids
**Cortisol**	1.118	1.060	$3.03\ 10^{-2}$	1.557	Lipids: Fatty acids-Steroids
**N-Acetylputrescine**	1.114	1.373	$2.29\ 10^{-2}$	1.275	Organic acids
**Thiamine monophosphate**	1.104	-0.904	$6.06\ 10^{-2}$	0.747	Vitamins-Cofactors-Hormones

**PC(O-18:2(9Z,12Z)/2:0)**	1.095	-0.842	$7.36\ 10^{-2}$	0.759	Phospholipids
**L-Arginine**	1.087	1.338	>0.1	1.163	Amino acids-Derivatives
**4-Trimethylammoniobutanoic acid**	1.067	-0.927	$4.71\ 10^{-2}$	0.825	Lipids: Fatty acids-Steroids
**L-Threonine**	1.064	1.579	>0.1	1.190	Amino acids-Derivatives
**Palmitic acid**	1.042	-0.829	$1.91\ 10^{-2}$	0.677	Lipids: Fatty acids-Steroids
**Hippuric acid**	1.035	-0.747	$3.29\ 10^{-2}$	0.494	Amino acids-Derivatives
**Ketoleucine**	1.029	0.799	$7.47\ 10^{-2}$	0.883	Amino acids-Derivatives
**L-Dopa**	1.011	1.360	$4.54\ 10^{-2}$	1.273	Vitamins-Cofactors-Hormones
**Valerolactone**	1.007	-0.823	$3.27\ 10^{-2}$	0.859	Others
**PC(14:0/20:1(11Z))**	1.005	0.682	>0.1	1.061	Phospholipids
**PC(14:0/22:1(13Z))**	1.000	0.760	>0.1	1.088	Phospholipids
**Adenosine monophosphate**	0.998	0.823	>0.1	2.181	Nucleotides-Nucleosides-Derivatives
**Urocanic acid**	0.991	-0.847	$4.35\ 10^{-2}$	0.448	Amino acids-Derivatives
**PC(18:1(11Z)/22:6(4Z,7Z,10Z,13Z,16Z,19Z))**	0.985	-0.876	>0.1	0.443	Phospholipids
**Succinic acid**	0.967	-0.785	$7.45\ 10^{-2}$	0.499	Organic acids
**NEPSILON,NEPSILON,NEPSILON-TRIMET**	0.956	0.574	>0.1	1.139	Others
**LysoPC(18:2(9Z,12Z))**	0.953	-0.628	>0.1	0.895	Phospholipids
**N-Methyltryptamine**	0.945	0.730	>0.1	1.270	Aromatic compounds-Amines
**Vanillylmandelic acid**	0.941	-0.721	$8.78\ 10^{-2}$	0.376	Aromatic compounds-Amines
**Pantothenic acid**	0.927	-0.614	$5.47\ 10^{-2}$	0.852	Vitamins-Cofactors-Hormones
**Serotonin**	0.913	0.475	>0.1	1.356	Amino acids-Derivatives
**N-Acetylmethionine**	0.911	-1.832	$3.96\ 10^{-2}$	0.617	Amino acids-Derivatives
**Picolinic acid**	0.908	0.685	>0.1	0.818	Amino acids-Derivatives
**SM(d18:1/18:0)**	0.908	0.418	>0.1	1.204	Lipids: Fatty acids-Steroids
**PC(O-16:1(11Z)/2:0)**	0.894	0.671	$9.09\ 10^{-2}$	1.193	Phospholipids
**PC(16:0/22:5(4Z,7Z,10Z,13Z,16Z))**	0.882	0.371	>0.1	0.989	Phospholipids
**L-Serine**	0.874	0.978	>0.1	1.190	Amino acids-Derivatives
**Pyroglutamic acid**	0.873	-0.552	$4.63\ 10^{-2}$	0.845	Amino acids-Derivatives
**LysoPC(16:0)**	0.867	0.491	>0.1	1.112	Phospholipids
**Glyceric acid**	0.861	0.784	>0.1	1.043	Sugar
**L-Methionine**	0.855	0.726	>0.1	1.153	Amino acids-Derivatives
**Gluconic acid**	0.848	-0.663	$5.10\ 10^{-2}$	0.743	Sugar
**Tetradecanoylcarnitine**	0.841	-0.581	>0.1	0.755	Lipids: Fatty acids-Steroids
**PC(14:1(9Z)/22:2(13Z,16Z))**	0.835	0.455	>0.1	0.907	Phospholipids

**Caproic acid**	0.832	-0.459	>0.1	0.887	Lipids: Fatty acids-Steroids
**Diethanolamine**	0.831	0.794	>0.1	1.266	Aromatic compounds-Amines
**Citric acid**	0.818	0.706	>0.1	1.928	Organic acids
**Spermidine**	0.801	0.648	>0.1	1.012	Aromatic compounds-Amines
**Diaminopimelic acid**	0.782	0.591	$8.81\ 10^{-2}$	1.339	Amino acids-Derivatives
**Menadione**	0.773	0.516	>0.1	1.769	Vitamins-Cofactors-Hormones
**Isocitric acid**	0.748	0.607	>0.1	1.677	Organic acids
**Indoleacetic acid**	0.739	0.397	>0.1	1.049	Aromatic compounds-Amines
**Orotic acid**	0.736	-0.332	>0.1	0.739	Nucleotides-Nucleosides-Derivatives
**Glucose 1-phosphate**	0.733	0.683	>0.1	1.525	Sugar
**1-Aminocyclopropanecarboxylic acid**	0.722	1.183	>0.1	1.090	Amino acids-Derivatives
**5-Hydroxylysine**	0.721	2.195	>0.1	1.119	Amino acids-Derivatives
**(2E,4E)-2,4-Hexadienoic acid**	0.715	0.230	>0.1	1.053	Lipids: Fatty acids-Steroids
**3-Aminoisobutanoic acid**	0.701	0.527	>0.1	1.023	Amino acids-Derivatives
**Glucose 6-phosphate**	0.699	0.552	>0.1	2.734	Sugar
**Malic acid**	0.695	-0.229	>0.1	0.913	Organic acids
**Galactitol**	0.679	-0.257	>0.1	0.844	Sugar
**trans-Cinnamate**	0.673	0.743	>0.1	1.090	Aromatic compounds-Amines
**Dethiobiotin**	0.665	0.280	>0.1	1.175	Lipids: Fatty acids-Steroids
**4-Acetamidobutanoic acid**	0.652	-0.365	>0.1	0.694	Organic acids
**Fumaric acid**	0.646	-0.172	>0.1	0.914	Organic acids
**Phosphocreatine**	0.621	0.454	>0.1	2.010	Amino acids-Derivatives
**Decanoylcarnitine**	0.617	0.496	>0.1	1.151	Lipids: Fatty acids-Steroids
**Riboflavin**	0.556	-0.156	>0.1	0.787	Vitamins-Cofactors-Hormones
**Deoxycholic acid**	0.466	0.357	>0.1	1.076	Lipids: Fatty acids-Steroids
**2-Hydroxypyridine**	0.440	-0.646	>0.1	0.977	Nucleotides-Nucleosides-Derivatives
**Taurine**	0.392	0.313	>0.1	1.028	Organic acids

Table S3

Elements only in serum :		
	p-value	Fold Change
3-(2-Hydroxyphenyl)propanoic acid	$1.84\ 10^{-3}$	1.821
3-Hydroxybutyric acid	$2.84\ 10^{-2}$	1.379
3-Methylhistamine	$2.77\ 10^{-2}$	0.731
3-Methylhistidine	$2.19\ 10^{-3}$	0.768
Allantoin	>0.05	0.885
Alpha-Tocopherol	$2.49\ 10^{-2}$	1.203
Azelaic acid	$4.63\ 10^{-2}$	0.756
Cholesteryl acetate	$1.10\ 10^{-3}$	1.929
Cholic acid	$3.19\ 10^{-5}$	20.144
Citramalic acid	$3.74\ 10^{-3}$	1.355
Corticosterone	>0.05	1.488
Deoxycytidine	$1.96\ 10^{-3}$	0.671
D-Glyceraldehyde-3-phosphate	>0.05	
Dodecanoylcarnitine	$1.74\ 10^{-2}$	1.969
Glucosamine 6-phosphate	$3.09\ 10^{-2}$	0.738
Glycerophosphocholine	$2.54\ 10^{-4}$	0.425
Glycocholic acid	$4.92\ 10^{-3}$	21.090
Guanidoacetic acid	$9.68\ 10^{-5}$	2.290
Indole-3-methyl acetate	$1.72\ 10^{-3}$	0.421
Indoxyl sulfate	$3.61\ 10^{-3}$	0.539
L-Acetylcarnitine	$6.81\ 10^{-5}$	1.531
L-Asparagine	$1.26\ 10^{-2}$	1.288
L-Glutamine	$1.96\ 10^{-2}$	1.186
L-Histidine	$1.89\ 10^{-2}$	1.164
Linoleic acid	>0.05	0.778
L-Lysine	$6.95\ 10^{-4}$	0.780
L-Pipecolic acid	$1.86\ 10^{-3}$	0.748
L-Valine	$9.30\ 10^{-3}$	1.411
LysoPC(16:1(9Z))	$2.75\ 10^{-2}$	0.839
LysoPC(20:0)	$1.49\ 10^{-1}$	0.694
LysoPC(20:2(11Z,14Z))	$1.37\ 10^{-3}$	0.856
LysoPC(20:3(5Z,8Z,11Z))	$5.48\ 10^{-2}$	0.837
LysoPE(0:0/18:1(11Z))	$3.41\ 10^{-2}$	1.434
Malonate	>0.05	1.191
Methylglutaric acid	$4.79\ 10^{-2}$	0.835

**N-Acetyl-L-phenylalanine**	$3.28\ 10^{-2}$	1.412
**N-Acetylserotonin**	$4.57\ 10^{-5}$	0.377
**N-acetyltryptophan**	$1.52\ 10^{-4}$	2.990
**N-Alpha-acetyllysine**	$2.13\ 10^{-3}$	0.794
**Nicotinic acid**	$6.80\ 10^{-4}$	1.659
**O-Acetyl-L-carnitine**	$7.14\ 10^{-5}$	
**Ornithine**	$1.25\ 10^{-2}$	1.479
**Oxoglutaric acid**	$1.63\ 10^{-3}$	1.702
**PC(14:0/16:0)**	$3.45\ 10^{-5}$	1.566
**PC(15:0/16:0)**	$5.84\ 10^{-4}$	1.441
**PC(15:0/18:2(9Z,12Z))**	$9.01\ 10^{-2}$	1.031
**PC(15:0/20:1(11Z))**	$1.45\ 10^{-1}$	1.140
**PC(15:0/20:2(11Z,14Z))**	$1.18\ 10^{-4}$	1.521
**PC(16:0/16:0)**	$1.05\ 10^{-2}$	1.276
**PC(20:4(5Z,8Z,11Z,14Z)/22:6(4Z,7Z,10Z,13Z,16Z,19Z))**	$2.40\ 10^{-2}$	0.648
**PC(O-14:1/2:0)**	$4.68\ 10^{-2}$	0.908
**PC(O-18:4/2:0)**	$4.87\ 10^{-2}$	1.140
**PE(16:0/20:4(5Z,8Z,11Z,14Z))**	$3.91\ 10^{-4}$	2.151
**PE(20:4(5Z,8Z,11Z,14Z)/18:1(11Z))**	$1.42\ 10^{-3}$	3.428
**PE(22:6(4Z,7Z,10Z,13Z,16Z,19Z)/18:1(11Z))**	$3.24\ 10^{-3}$	3.738
**PE(O-16:1(1Z)/22:6(4Z,7Z,10Z,13Z,16Z,19Z))**	$1.57\ 10^{-2}$	1.720
**Phosphorylcholine**	$4.35\ 10^{-2}$	0.679
**Stearic acid**	$9.25\ 10^{-3}$	0.707
**trans-Ferulic acid**	>0.05	0.504
**Trigonelline**	$1.39\ 10^{-4}$	0.425

**Elements only in soleus :**		
	**p-value**	**Fold change**
**(2E,4E)-2,4-Hexadienoic acid**	>0.1	1.053
**1-Aminocyclopropanecarboxylic acid**	>0.1	1.090
**2-Hydroxypyridine**	>0.1	0.977
**3-Aminoisobutanoic acid**	>0.1	1.023
**3-Hydroxybenzoic acid**	$7.60\ 10^{-3}$	0.682
**4-Acetamidobutanoic acid**	>0.1	0.694
**5-Hydroxylysine**	>0.1	1.119
**Adenosine monophosphate**	>0.1	2.181
**Caproic acid**	>0.1	0.887
**Caprylic acid**	$6.04\ 10^{-3}$	2.065

**Cortisol**	3.03 10^{-2}	1.557
**Creatine**	1.56 10^{-2}	1.195
**Cytidine**	9.09 10^{-4}	0.803
**Decanoylcarnitine**	>0.1	1.151
**Deoxycholic acid**	>0.1	1.076
**Dethiobiotin**	>0.1	1.175
**Diaminopimelic acid**	8.81 10^{-2}	1.339
**Diethanolamine**	>0.1	1.266
**Galactitol**	>0.1	0.844
**Glucose 1-phosphate**	>0.1	1.525
**Glucose 6-phosphate**	>0.1	2.734
**Glyceric acid**	>0.1	1.043
**Guanine**	3.33 10^{-2}	0.778
**Guanosine**	2.63 10^{-3}	0.602
**Inosine**	1.81 10^{-2}	0.768
**L-Aspartic acid**	6.69 10^{-2}	0.555
**L-Dopa**	4.54 10^{-2}	1.273
**L-Homoserine**	>0.1	1.202
**LysoPC(18:2(9Z,12Z))**	>0.1	0.895
**Menadione**	>0.1	1.769
**Methylguanidine**	1.57 10^{-2}	1.399
**N-Acetylmethionine**	3.96 10^{-2}	0.617
**N-Acetylputrescine**	2.29 10^{-2}	1.275
**NEPSILON,NEPSILON,NEPSILON-TRIMET**	>0.1	1.139
**Pantothenic acid**	5.47 10^{-2}	0.852
**PC(14:0/22:1(13Z))**	>0.1	1.088
**PC(14:1(9Z)/22:2(13Z,16Z))**	>0.1	0.907
**PC(16:0/22:5(4Z,7Z,10Z,13Z,16Z))**	>0.1	0.989
**PC(16:1(9Z)/22:2(13Z,16Z))**	>0.1	1.095
**PC(18:1(11Z)/22:6(4Z,7Z,10Z,13Z,16Z,19Z))**	>0.1	0.443
**PC(21:0)**	>0.1	0.984
**Phosphocreatine**	>0.1	2.010
**Picolinic acid**	>0.1	0.818
**Pyroglutamic acid**	4.63 10^{-2}	0.845
**Riboflavin**	>0.1	0.787
**SM(d18:1/18:0)**	>0.1	1.204
**Spermidine**	>0.1	1.012
**Succinic acid**	7.45 10^{-2}	0.499
**Taurine**	>0.1	1.028

**trans-Cinnamate**	>0.1	1.090
**Valerolactone**	$3.27\ 10^{-2}$	0.859
**Vanillylmandelic acid**	$8.78\ 10^{-2}$	0.376

**Common elements in serum soleus :**	**Serum**		**Soleus**	
	**p-value**	**Fold Change**	**p-value**	**Fold Change**
**Biotin**	$8.85\ 10^{-4}$	2.284	$6.45\ 10^{-3}$	0.576
**4-Trimethylammoniobutanoic acid**	$1.15\ 10^{-4}$	1.553	$4.71\ 10^{-2}$	0.825
**Citric acid**	$4.38\ 10^{-2}$	1.187	>0.1	1.928
**Cytosine**	$3.10\ 10^{-4}$	0.601	$3.18\ 10^{-3}$	0.754
**D-Glucose**	>0.05	0.806	$4.11\ 10^{-2}$	1.329
**Fumaric acid**	$4.96\ 10^{-2}$	1.424	>0.1	0.914
**Gluconic acid**	$3.22\ 10^{-2}$	0.575	$5.10\ 10^{-2}$	0.743
**Hippuric acid**	$3.50\ 10^{-6}$	0.056	$3.29\ 10^{-2}$	0.494
**Indoleacetic acid**	$1.77\ 10^{-4}$	2.569	>0.1	1.049
**Isocitric acid**	$4.12\ 10^{-2}$	1.196	>0.1	1.677
**Ketoleucine**	$7.24\ 10^{-4}$	0.474	$7.47\ 10^{-2}$	0.883
**L-Arginine**	$7.97\ 10^{-3}$	0.729	>0.1	1.163
**L-Lactic acid**	>0.05	0.928	$1.19\ 10^{-2}$	1.158
**L-Malic acid**	$5.88\ 10^{-3}$	1.634	>0.1	0.913
**L-Methionine**	>0.05	0.949	>0.1	1.153
**L-Serine**	>0.05	1.003	>0.1	1.190
**L-Threonine**	>0.05	0.964	>0.1	1.190
**LysoPC(16:0)**	$5.30\ 10^{-3}$	0.910	>0.1	1.112
**LysoPC(18:0)**	$4.35\ 10^{-3}$	0.857	>0.1	1.259
**N-Methyltryptamine**	$1.45\ 10^{-3}$	0.323	>0.1	1.270
**Orotic acid**	>0.05	0.753	>0.1	0.739
**Palmitic acid**	$3.56\ 10^{-2}$	0.687	$1.91\ 10^{-2}$	0.677
**PC(14:0/20:1(11Z))**	$3.86\ 10^{-1}$	1.105	>0.1	1.061
**PC(18:1(9Z)/18:1(9Z))**	$3.75\ 10^{-3}$	1.289	>0.1	1.339
**PC(O-14:1/2:0)**	$3.77\ 10^{-2}$	0.828	$3.99\ 10^{-4}$	0.852
**PC(O-16:1(11Z)/2:0)**	$3.89\ 10^{-2}$	0.874	$9.09\ 10^{-2}$	1.193
**PC(O-18:2(9Z,12Z)/2:0)**	$1.16\ 10^{-3}$	0.843	$7.36\ 10^{-2}$	0.759
**Platelet activating factor**	$4.50\ 10^{-3}$	0.857	>0.1	1.303
**Propionylcarnitine**	$8.34\ 10^{-3}$	1.520	$9.18\ 10^{-3}$	1.498
**Serotonin**	$8.87\ 10^{-6}$	0.476	>0.1	1.356
**Tetradecanoylcarnitine**	$2.89\ 10^{-4}$	2.052	>0.1	0.755
**Thiamine monophosphate**	$2.17\ 10^{-3}$	1.809	$6.06\ 10^{-2}$	0.747
**Uracil**	$7.39\ 10^{-3}$	0.706	$6.32\ 10^{-3}$	0.659

**Uric acid**	3.60 10^{-2}	0.759	4.43 10^{-3}	0.777
**Uridine**	1.82 10^{-3}	0.671	1.10 10^{-2}	0.662
**Urocanic acid**	>0.05	1.037	4.35 10^{-2}	0.448
